# Neuritin: a multifaceted neuroprotective factor with emerging applications for neurodegeneration

**DOI:** 10.3389/fnins.2025.1698598

**Published:** 2025-12-05

**Authors:** Kai L. Mongan, Tasneem P. Sharma

**Affiliations:** 1 Department of Ophthalmology, Indiana University School of Medicine, Indianapolis, IN, United States; 2 Department of Pharmacology and Toxicology, Indiana University School of Medicine, Indianapolis, IN, United States

**Keywords:** neuritin, neuroprotection, synaptic plasticity, retinal ganglion cells, neurodegeneration

## Abstract

Neuritin is a conserved, activity-regulated gene encoding a glycosylphosphatidylinositol-anchored protein, crucial for neural development, synaptic plasticity, and neuroprotection. Identified via activity-dependent gene screening in the rat hippocampus, neuritin promotes neurite outgrowth, dendritic arborization, and synaptic maturation with neural activity. In this review, we summarize recent findings regarding neuritin’s signaling pathways, neuroprotective, neuroregenerative, and neuromodulatory properties, with a focus on its therapeutic potential to counter neurodegeneration in various conditions such as glaucoma, Alzheimer’s disease, stroke, diabetic neuropathy, and neuropsychiatric disorders. Additionally, recent studies reveal roles in immunoregulation, angiogenesis, and cancer biology, highlighting neuritin as a versatile signaling molecule with broad therapeutic implications.

## 1 Introduction

The human nervous system contains about 86 billion neurons connected through trillions of synapses, creating complex networks that support cognition, behavior, and physiological balance (Azevedo et al., 2009). Central to the proper operation of these neural networks is synaptic plasticity, the ability of synapses to strengthen or weaken over time in response to changes in their activity (Citri and Malenka, 2008). This essential property also allows for neuronal repair and functional recovery post injury (Nagappan et al., 2020).

Activity-dependent synaptic plasticity involves changes in gene expression, protein synthesis, and structural modifications at synapses (West and Greenberg, 2011). These processes are driven by immediate early genes, neurotrophic factors, and activity-regulated proteins that convert neural activity into lasting synaptic changes (Flavell and Greenberg, 2008). Neurotrophic factors are essential for neural development, survival, and plasticity, such as brain-derived neurotrophic factor (BDNF) and nerve growth factor (NGF), which are known for promoting neuronal survival, axon guidance, and synaptic plasticity (Huang and Reichardt, 2001; Chao, 2003). Ongoing research into novel neurotrophic molecules continues to expand our understanding of the complex signaling networks regulating neural functions.

The central nervous system’s remarkable ability for structural and functional remodeling extends beyond development into adulthood, supporting learning, memory consolidation, and recovery from injury (Pascual-Leone et al., 2005). This neuroplasticity is driven by activity-dependent changes in synaptic strength, dendritic spine morphology, and neural connectivity patterns (Holtmaat and Svoboda, 2009). Understanding the molecular mechanisms behind these processes has significant implications for developing neuroprotective treatments for neurodegenerative diseases and psychiatric disorders.

Neurodegenerative diseases are a rising global issue, affecting millions. Diseases like Alzheimer’s (AD), hearing loss, and stroke harm neural circuits, causing decline and disability (Mattson, 2000). Glaucoma is the leading cause of irreversible blindness, marked by retinal ganglion cell (RGC) death and optic nerve damage (Weinreb et al., 2014). Current treatments mainly manage symptoms, not the root cause (Jellinger, 2010; Mangialasche et al., 2010). Finding neuroprotective factors and pathways could lead to treatments that save neurons and prevent damage.

Neuritin (NRN1) is a signaling molecule important for neural development, synaptic plasticity, and protecting neurons (Nedivi et al., 1993). Initially discovered in the search of activity-dependent genes linked to neural plasticity. To uncover these genes, researchers developed a strategy to find genes related to plasticity by creating a cDNA library from mRNA in the rat hippocampus after kainate-induced neural activation, which stimulates synaptic activity and plasticity. Genes upregulated here were hypothesized to likely play a role in activity-dependent neural plasticity and were termed candidate plasticity-related genes (CPGs) (Nedivi et al., 1993). Among these, *Cpg15* stood out as a gene of special interest after initial functional and molecular studies showed it is crucial for encouraging neurite growth and dendritic branching, inspiring its name NRN1 (Corriveau et al., 1999). Structurally, the protein has a conserved 142-amino acid sequence, an N-terminal signal peptide, and a C-terminal GPI anchor, appearing to function as a tightly regulated, membrane-associated effector in CNS development, remodeling, and plasticity (Nedivi et al., 1993; Naeve et al., 1997; Corriveau et al., 1999).

This review assesses the current knowledge of NRN1’s molecular mechanisms, physiological functions, and therapeutic potential to safeguard against neurodegeneration. We explore the complex cellular regulation of NRN1, its roles in neural development and plasticity, and emerging evidence of its involvement in disease pathogenesis. Additionally, we explore the complex signaling networks through which it functions. Lastly, we provide a detailed review of the translational implications of NRN1 research linking neurobiology and therapy, emphasizing its potential as a therapeutic target for various challenging neurological conditions, with particular focus on its promising applications in ophthalmology and neuroprotection.

## 2 Molecular mechanisms of neuritin regulation

### 2.1 Activity-dependent signaling pathways

*NRN1* has emerged as a key regulator of neuronal development, synaptic plasticity, and neuroprotection. *NRN1* exhibits sophisticated regulatory mechanisms that respond to diverse cellular stimuli while maintaining a baseline expression level independent of neuronal activity. Understanding these regulatory pathways is crucial for elucidating the molecular basis of neural plasticity and identifying potential therapeutic targets for neurodevelopmental and neurodegenerative disorders.

The transcriptional regulation of *NRN1* is closely connected to excitatory neurotransmission, with multiple glutamate receptor types contributing to its induction. Studies in rats show that *Nrn1* transcription is linked to excitatory signals of N-methyl-D-aspartate (NMDA) receptor activation, α-Amino-3-hydroxy-5-methyl-4-isoxazolepropionic acid (AMPA) receptor activity, and glutamate analog treatments, all of which boost *Nrn1* mRNA levels (Naeve et al., 1997). This responsiveness indicates that *Nrn1* acts as a convergence point for excitatory synaptic activity. Kainate-induced neuronal activity represents another key pathway for *Nrn1* induction, demonstrating that excitatory signaling through different receptor systems influences *Nrn1* expression. Pharmacological studies further clarify this specificity, showing that various stimuli like KCl depolarization and BDNF treatment can effectively upregulate *Nrn1* (Nedivi et al., 1993; Naeve et al., 1997).

Calcium influx serves as a critical mediator of activity-dependent *Nrn1* expression. Voltage-gated calcium channel blockers reveal that L-type channels are crucial for KCl-induced *Nrn1* induction. Nifedipine inhibits *Nrn1*, underscoring calcium influx. Blocking calcium/calmodulin-dependent protein kinase II (CaMKII) with KN-62 also reduces *Nrn1*, highlighting calcium’s role as a secondary messenger (Naeve et al., 1997). In rat hippocampal cultures, *Nrn1* expression persists even when NMDA and AMPA receptors are blocked, indicating voltage-gated channels and calcium signaling can independently regulate *Nrn1*, when glutamatergic transmission is pharmacologically blocked (Naeve et al., 1997). Further research with kinase inhibitors expanded our understanding of calcium-responsive pathways, revealing that both CaMK and mitogen-activated protein kinase (MAPK) pathways are essential for optimal *Nrn1* expression (Fujino et al., 2003).

BDNF is another regulator of *Nrn1*, operating through mechanisms distinct from calcium/calmodulin signaling. Its effect expression can be blocked by TrkB receptor antagonism, confirming the involvement of the canonical BDNF–TrkB signaling pathway. Interestingly, KN-62 does not inhibit BDNF-driven *Nrn1* induction, implying that BDNF–TrkB activates *Nrn1* through calcium-independent pathways (Naeve et al., 1997). This mechanistic distinction highlights the diversity of signaling pathways that converge on the regulation of *Nrn1* expression.

### 2.2 Activity-independent expression patterns

The convergence of CaMK and MAPK signaling pathways parallels established mechanisms of immediate early gene activation. This transcriptional control mechanism bears striking similarities to the regulation of the c-fos gene, where both CaMK and MAPK pathways promote transcription through the cAMP response element-binding protein. At the same time, MAPK additionally activates the transcription factor Elk-1 (Xia et al., 1996). Although the upstream regulatory sequence of *NRN1* lacks an identifiable Elk-1 binding site, other transcription factors likely fulfill analogous roles in mediating MAPK-dependent transcriptional activation. The identification of these alternative transcription factors represents an important area for future investigation.

Sequence analysis of the *Nrn1* promoter region has revealed the presence of two sequences with high similarity to cAMP-responsive elements (CREs). Functional characterization through site-directed mutagenesis has provided crucial insights into the individual contributions of these regulatory sequences. Remarkably, mutations introduced at different CRE sites produced opposing effects on gene expression, with some mutations increasing transcriptional activity while others decreased it (Fujino et al., 2003). These findings demonstrate that CRE sites exert both positive and negative regulatory control over *Nrn1* transcription, suggesting a complex regulatory network that fine-tunes gene expression in response to cellular conditions. Chromatin immunoprecipitation assays conducted in mouse cortical cultures have confirmed cAMP-response element binding protein binding to these CRE promoter elements, providing direct evidence that cAMP-generating pathways dynamically regulate *Nrn1* expression (Fujino et al., 2003). These findings suggest that the regulation of *Nrn1* extends beyond activity-dependent mechanisms to include constitutive, activity-independent expression patterns. This dual regulatory system reflects the multifaceted roles of *Nrn1* in neural development and function. Activity-independent *Nrn1* expression occurs during early embryonic development, preceding circuit formation and axon migration, which emphasizes *Nrn1’s* fundamental role in early neural development processes (Nedivi et al., 2001; Putz et al., 2005).

Investigations using embryonic rat brain tissue have proposed a functional differentiation between activity-independent and activity-dependent *Nrn1* expression. According to this model, activity-independent expression serves primarily as an anti-apoptotic factor during early neural development. In contrast, subsequent activity-dependent expression functions as a growth and differentiation factor during later developmental stages and synaptic plasticity (Putz et al., 2005). This temporal and functional segregation suggests that *Nrn1* expression is dynamically regulated to meet the changing requirements of developing and mature neural circuits. The anti-apoptotic function during early development would be crucial for neuronal survival during the period of programmed cell death, while the growth-promoting functions would support axon extension, dendritic elaboration, and synaptic formation.

Adding another layer of complexity, two distinct forms of the NRN1 protein are expressed in the CNS. These isoforms differ in their subcellular localization and functional properties, reflecting specialized roles in neural development and neuroprotection. The membrane-bound form of NRN1, anchored to the cell surface through GPI linkage, positions it perfectly to participate in cell–cell interactions and membrane-associated signaling processes. In contrast, the soluble variant after cleavage from the GPI anchor can diffuse through the extracellular space and potentially act on distant cellular targets (Nedivi et al., 1998; Javaherian and Cline, 2005; Putz et al., 2005). These structural differences translate into distinct functional properties. The soluble form of NRN1 has been noted for its unique ability to inhibit apoptosis, supporting neuronal survival during development and in response to cellular stress (Putz et al., 2005). Conversely, the membrane-bound form promotes axon growth and extension, facilitating the establishment of neural connectivity (Nedivi et al., 1998; Javaherian and Cline, 2005). This functional specialization suggests that alternative processing or differential regulation of NRN1 isoforms provides a mechanism for tailoring cellular responses to specific developmental requirements or environmental conditions.

The complex regulatory mechanisms governing *NRN1* expression highlight its central role in neural development and plasticity. The integration of multiple signaling pathways, including glutamatergic transmission, calcium-dependent cascades, and growth factor signaling, positions *NRN1* as a key mediator of activity-dependent neural modifications. The existence of both activity-dependent and activity-independent regulatory mechanisms, combined with functionally distinct protein isoforms, provides a sophisticated system for fine-tuning neural development and plasticity responses. Understanding these mechanisms has significant implications for developing therapeutic interventions for neurodevelopmental disorders, neurodegenerative conditions, and nervous system injuries.

*NRN1* represents a paradigmatic example of how neural activity regulates gene expression to support both developmental processes and ongoing synaptic plasticity. The multi-layered regulatory mechanisms that encompass transcriptional control, post-translational modifications, and alternative isoform expression provide the nervous system with sophisticated tools for adapting to changing functional demands. Continued investigation of these regulatory networks will enhance our understanding of neural plasticity mechanisms and potentially reveal new therapeutic targets for treating neurological conditions. Future research directions should focus on identifying the specific transcription factors that mediate MAPK-dependent *NRN1* activation, characterizing the mechanisms that determine the balance between membrane-bound and soluble NRN1 isoforms, and investigating how dysregulation of these pathways contributes to neurological disorders.

### 2.3 NRN1: organ expression and cellular location

Comprehensive analysis of *Nrn1* mRNA expression in rat tissues has demonstrated that its distribution is regionally enriched in brain areas associated with high synaptic plasticity. Expression is particularly prominent in the hippocampus, olfactory bulb, and Purkinje cells of the cerebellum, regions known for their roles in learning, memory, and sensorimotor coordination. Further *in situ* hybridization and immunohistochemical studies revealed additional expression in the cerebral cortex, thalamus, and cerebellum, with exceptionally high levels localized to the dentate gyrus of the hippocampus, tenia tecta, olfactory bulb, retinal ganglion cells (RGCs), and the optic nerve layer of the superior colliculus (Naeve et al., 1997). This anatomically restricted expression pattern aligns with the proposed role of NRN1 as a mediator of activity-dependent structural remodeling within the CNS.

In contrast, a survey of *NRN1* expression in human tissues revealed a broader distribution beyond the nervous system. While the highest expression levels were consistently observed in the brain, *NRN1* transcripts were also detected at moderate levels in skeletal muscle, lung, thymus, pancreas, placenta, liver, and heart. In comparison, lower expression levels were reported in the spleen, testes, ovaries, and small intestine, indicating tissue-specific regulation that may reflect distinct functional roles across organ systems (Dong et al., 2018).

## 3 NRN1: neuronal and other functions

**Figure 1:**
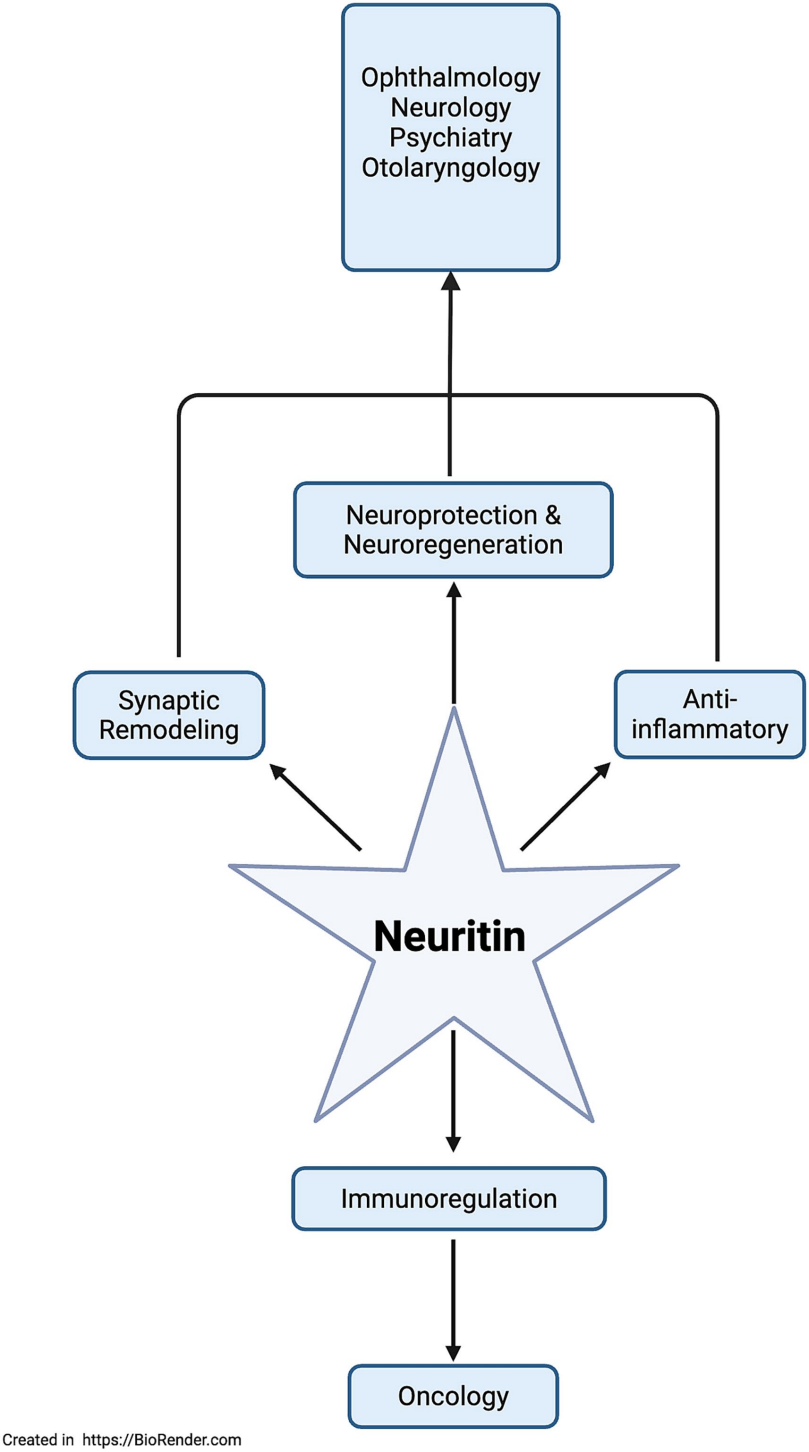
Flowchart of NRN1’s function paired with clinical specialties of research interest.

NRN1’s functions have yet to be investigated in clinical trials, but preclinical research has established NRN1’s role as a neurotrophic factor with neuroprotective and regenerative properties that enhance neurite outgrowth and synaptic maturation through antiapoptotic and anti-inflammatory mechanisms. NRN1 research also indicates an emerging role in immunoregulation and angiogenesis. Figure 1 depicts NRN1, and its functional role linked to clinically relevant specialties.

### 3.1 NRN1 stimulates neurite outgrowth

At the cellular level, NRN1 expression is predominantly localized to neuronal cell bodies and neurite processes, including dendrites and axons (Gomes et al., 2017; Lu et al., 2017). This subcellular distribution is consistent with its established role in promoting neurite outgrowth and synapse formation. Immunofluorescence studies conducted in SH-SY5Y neuroblastoma cells revealed that NRN1 localizes specifically to lipid raft microdomains on the plasma membrane, which are specialized membrane compartments involved in signal transduction and protein trafficking (Dong et al., 2018). These results highlight the importance of membrane localization in facilitating NRN1’s activity-dependent signaling functions.

Interestingly, the subcellular localization of NRN1 is dynamic and can be influenced by the neurochemical environment. In models of peripheral nerve injury, NRN1 has been shown to undergo a redistribution from predominantly somatic localization to axonal compartments, suggesting a role in axonal regeneration and repair following neural damage (Merianda et al., 2013). This plasticity in localization further supports structural neuronal remodeling, both under physiological and pathological conditions.

Given *Nrn1’s* expression during development and its sustained postnatal presence in neuronal cell bodies and axons, it is likely that NRN1 plays a vital role in maintaining neuronal differentiation and function. Initially identified for its ability to induce pronounced morphological changes in rat hippocampal and cortical neurons, NRN1 has been shown to enhance dendritic complexity and synapse formation and at the cellular level, NRN1-treated neurons exhibit well-differentiated cell bodies and extensions, in contrast to untreated cells, which display flattened cell bodies and disorganized, broadly distributed extensions characteristic of poorly differentiated neurons (Cantallops et al., 2000; Javaherian and Cline, 2005).

NRN1 also significantly influences neurite outgrowth, as demonstrated in several studies (Marron et al., 2005; Li et al., 2015; Zhang et al., 2018). This role was further substantiated by RNA interference experiments, where silencing of *Nrn1* abolished nerve growth factor (NGF)-induced neurite outgrowth (Karamoysoyli et al., 2008). Importantly, NRN1-mediated axonal growth is neuron-type specific: it promotes outgrowth in projection neurons but does not affect interneurons (Nedivi et al., 1998). This selectivity suggests distinct molecular mechanisms governing axonal development across different neuronal subtypes.

In addition to promoting neuronal growth, NRN1 appears to influence neuronal migration. Immortalized migrating neurons derived from rats express significantly higher levels of NRN1 compared to non-migrating cells, and *Nrn1* overexpression enhances migration, while knockdown impairs it (Zito et al., 2014). These findings suggest that NRN1 may facilitate the navigation of neuronal processes through their microenvironment and aid in establishing appropriate synaptic connections.

### 3.2 NRN1 and its role in synaptic maturation, stabilization, and transmission

Continued research into the neuronal functions of NRN1 has revealed its critical involvement in coordinating axonal development with synaptic maturation processes. Early studies demonstrated that *nrn1* expression correlates with the simultaneous growth of axonal arbors and the maturation of synapses, suggesting a synchronized developmental program (Cantallops et al., 2000; Javaherian and Cline, 2005). In Xenopus optic tectal neurons expressing *nrn1*, axonal arborization proceeded concurrently with synaptic strengthening, evidenced by the recruitment of functional AMPA-type glutamate receptors. The incorporation of these receptors into the postsynaptic density signifies synaptic potentiation, a crucial component of long-term synaptic plasticity mechanisms, such as long-term potentiation. This indicates that NRN1 actively participates in reinforcing synaptic connections during both development and learning processes (Cantallops et al., 2000).

Further investigations into NRN1’s role in synaptic stabilization employed a pioneering knockout (KO) mouse model developed by Nedivi and colleagues, which lacked the *Nrn1* gene. Their findings demonstrated that NRN1 is vital for maintaining active synapses on dendritic spines. Dendrites within the visual cortex of *Nrn1* KO mice exhibited a significant reduction in dendritic spine density compared to wild-type (WT) controls, indicating that loss of NRN1 impairs the stabilization and maintenance of excitatory synapses (Fujino et al., 2011). This deficit underscores NRN1’s crucial role in maintaining synaptic structures essential for effective neuronal communication.

Building on these findings, it was shown that sensory experience influences NRN1-driven synaptic stabilization. By using visual stimulation protocols to induce neuronal activity, they discovered that NRN1 mimics experience-dependent plasticity by specifically stabilizing excitatory synapses. This process involves the recruitment of the synaptic scaffolding protein PSD-95, which is instrumental in organizing postsynaptic receptor complexes and maintaining synaptic strength. These results illuminate the mechanistic pathway through which NRN1 facilitates experience-dependent synaptic refinement and stabilization, highlighting its importance in adaptive neural circuitry (Fujino et al., 2011).

In addition to its stabilizing functions, NRN1 has a profound influence on synaptic transmission. Electrophysiological recordings combined with high-performance liquid chromatography analyses have demonstrated that NRN1 treatment enhances presynaptic glutamate release and increases spontaneous miniature excitatory postsynaptic current frequency in neurons of the medial prefrontal cortex in mice (Lu et al., 2017). These findings suggest that NRN1 elevates overall synaptic activity by promoting neurotransmitter release. Notably, silencing *Nrn1* expression reverses these effects, impairing synaptic efficacy, which confirms its mechanistic role in modulating synaptic transmission strength (Lu et al., 2017). This bidirectional relationship between *Nrn1* expression and synaptic function illustrates its direct regulatory role in neural communication.

### 3.3 NRN1: neuroregenerative and neuroprotective capabilities

The well-established roles of NRN1 in promoting neuronal differentiation, neurite extension, and axon growth have led to increasing interest in its therapeutic potential for neuroregeneration and neuroprotection. Numerous studies have demonstrated that neuritin is not only involved in developmental processes but also plays a pivotal role in neuronal repair and survival in both acute and chronic injury contexts.

Initial evidence for NRN1’s regenerative capabilities emerged from spinal cord injury model in rats. In a study profiling gene expression in regenerating dorsal root ganglia, *Nrn1* was identified as part of a gene cluster including Attractin and Microtubule-associated protein 1A, whose coordinated expression was associated with axonal plasticity and regeneration (Di Giovanni et al., 2005). Notably, *Nrn1* displayed a biphasic expression pattern, being initially downregulated after injury and subsequently showing strong upregulation during the regenerative phase. This dynamic response suggested that NRN1 may act as a key switch in the transition from injury-induced suppression to repair initiation (Di Giovanni et al., 2005). Follow-up studies confirmed that exogenous delivery of NRN1 following spinal cord injury significantly enhanced axonal regeneration and improved locomotor function in affected limbs (Gao et al., 2016).

The neuroregenerative potential of NRN1 has also been validated in models of peripheral neuropathy. In diabetic rats, treatment with NGF resulted in increased expression of NRN1, which correlated with improved peripheral nerve regeneration (Karamoysoyli et al., 2008). These findings support a model in which NGF-induced *Nrn1* expression plays a critical role in the restoration of neuronal integrity under metabolic stress conditions.

Given NRN1’s strong expression in the retina and optic nerve researchers have applied it in models of optic nerve injury to evaluate its effects on RGC survival and regeneration (Lee and Nedivi, 2002; Picard et al., 2014). In an optic nerve crush model, adenovirus-mediated overexpression of NRN1 resulted in marked enhancement of RGC survival, increased neurite outgrowth, and preservation of visual function (Sharma et al., 2015). These functional improvements were accompanied by evidence of active axonal transport across the lesion site, as well as increased expression of regeneration-associated markers such as Gap43 and the RGC-specific marker, RNA binding protein with multiple splicing (Sharma et al., 2015). A complementary study using optical coherence tomography demonstrated that NRN1 administration mitigated the post-injury thinning of the ganglion cell complex, further reinforcing its protective effects on RGC structure and function (Azuchi et al., 2018).

NRN1’s neuroprotective properties are not limited to structural support but also include the molecular inhibition of neuronal cell death. Multiple injury models, including optic nerve crush, traumatic brain injury, ischemic stroke, and hyperglycemia-induced neurotoxicity, have consistently demonstrated NRN1’s ability to reduce neuronal apoptosis (Sharma et al., 2015; Liu et al., 2018; Yan et al., 2018; Xu et al., 2023). Across these models, NRN1 was shown to suppress the intrinsic (mitochondrial) apoptotic pathway by increasing levels of the anti-apoptotic protein Bcl-2 and reducing the activation of executioner caspases, particularly caspase-3 and -9.

In addition to its anti-apoptotic effects, recent findings suggest that NRN1 also confers protection against ferroptosis, a form of regulated cell death driven by iron-dependent lipid peroxidation. In this context, NRN1 was found to enhance the activity of NAD + kinase, which in turn increased the intracellular pool of NADPH, a key antioxidant and suppressor of ferroptosis induced cell death. By modulating this redox pathway, NRN1 helps preserve neuronal viability under conditions of oxidative stress (Song et al., 2025).

Taken together, these findings underscore NRN1’s multifaceted role as a neurotrophic factor that not only guides synaptic development and plasticity but also confers robust neuroregenerative and neuroprotective benefits across various injury paradigms. Its ability to promote axonal regrowth, support synaptic function, and prevent neuronal death through multiple cellular pathways highlights its potential as a therapeutic target for neurodegenerative diseases, traumatic injury, and neuroinflammatory conditions.

### 3.4 NRN1: emerging roles in immunoregulation and angiogenesis

While NRN1 is well-established as a critical regulator of neuronal development, synaptic plasticity, and neuroprotection, emerging evidence has identified its involvement in physiological processes beyond the nervous system, particularly in immune regulation and angiogenesis.

Recent studies have elucidated an immunomodulatory role for NRN1 in the adaptive immune system, particularly in the function and differentiation of regulatory T cells (Tregs). NRN1 appears to regulate the metabolic state of T cells and promote the expansion and differentiation of Tregs toward a phenotype specialized for homing to peripheral tissues (Mahne et al., 2015; Gonzalez-Figueroa et al., 2021; Yu et al., 2024; Zhang et al., 2025). This tissue-adapted phenotype supports local immune tolerance and immune homeostasis. Further investigation has shown that NRN1 influences T cell energy, a state of non-responsiveness in T cells, and modulates autoantibody production through Treg-dependent pathways (Mahne et al., 2015). These findings position NRN1 as a potential therapeutic target in autoimmune disorders, where regulation of Treg function is often impaired. Additionally, transcriptomic profiling of NRN1-expressing Tregs has revealed a unique immunoregulatory gene signature that underscores its role in maintaining immune tolerance (Mahne et al., 2015). Together, these findings suggest NRN1 may play a role in immune regulation by shifting the balance of T cells toward the anti-inflammatory Treg phenotype, which reduces autoantibody production and inhibits inflammation in peripheral tissues such as the nervous system. This could have implications for autoimmune neurologic diseases, providing a potential new target for research in disease models of these disorders.

Parallels between NRN1’s role in guiding neuronal migration and its function in vascular biology have also been observed. Notably, NRN1 has been shown to promote endothelial cell migration. In a wound healing assay, treatment with NRN1 induced a dose-dependent increase in the migration of human umbilical vein endothelial cells, highlighting its potential pro-angiogenic properties (Han et al., 2011). *In vivo* studies using athymic nude mice demonstrated that xenografts overexpressing NRN1 exhibited markedly increased vascularization, as evidenced by elevated expression of the endothelial marker CD31 within tumor tissue. This suggests that NRN1 may stimulate angiogenesis by directly enhancing the function of endothelial cells and promoting vessel formation.

A subsequent study further confirmed NRN1’s role in vascular remodeling and tumor biology (Yang et al., 2021). Their work demonstrated that NRN1 not only promoted human umbilical vein endothelial cell migration but also enhanced tumor-associated angiogenesis *in vivo*, supporting its contribution to pathological neovascularization (Yang et al., 2021). These findings suggest that NRN1 may act as a molecular bridge between neural and vascular development, functioning in both contexts to direct cellular migration and structural remodeling.

These studies highlight the multifaceted functions of NRN1 across biological systems. Its emerging roles in immunoregulation and angiogenesis expand its relevance beyond neurobiology and underscore its potential as a target for therapeutic intervention in immune-mediated diseases, cancer, and vascular pathologies.

## 4 NRN1 signaling pathways: mechanisms and downstream targets

Research on NRN1 has primarily focused on its transcriptional regulation and roles in neuroregeneration and neuronal survival, while the cellular signaling pathways activated by NRN1 have received considerably less attention. This gap in understanding represents a critical frontier in neurobiology, as elucidating NRN1’s signaling mechanisms is essential for comprehending its diverse physiological functions and therapeutic potential. Recent advances have begun to illuminate the complex network of pathways through which NRN1 exerts its effects on neuronal excitability, growth, and survival.

### 4.1 Insulin receptor-mediated signaling

The initial indication that NRN1 functions as an intercellular signaling molecule emerged from pioneering work showing that NRN1 expression in one neuron could stimulate dendritic growth in adjacent neurons (Yao et al., 2018). This paracrine effect suggests that NRN1 operates beyond simple cell-autonomous mechanisms, potentially through direct interaction with cell surface receptors either with itself through homophilic binding or with distinct, yet-to-be-identified receptor systems. This discovery fundamentally shifted the understanding of NRN1 from a passive structural protein to an active signaling molecule capable of coordinating intercellular communication within neural networks.

Significant progress in elucidating NRN1’s signaling mechanisms via insulin receptor (IR) pathways has demonstrated that NRN1 treatment of rat cerebellar granule neurons increase transient outward potassium currents (IA) by upregulating Kv4.2, the primary subunit of voltage-gated potassium channels (Yao et al., 2012). These channels are essential regulators of neuronal excitability in the central nervous system, controlling the repolarization phase of action potentials and influencing firing patterns.

Given the established role of insulin in enhancing IA currents through Kv4.2-dependent mechanisms, the research team hypothesized that NRN1 might bind to and activate IR (Yao et al., 2012). This hypothesis gained substantial support when pharmacological blockade of IR completely prevented NRN1-induced increases in Kv4.2 expression and IA current density (Yao et al., 2012). This finding represents one of the most direct pieces of evidence for a specific receptor-mediated mechanism of NRN1 action.

To test the specificity of this interaction, researchers examined whether the structurally homologous insulin-like growth factor type 1 receptor (IGF-1R) might also mediate NRN1’s effects. Notably, pharmacological blockade of IGF-1R did not prevent NRN1-induced elevation of IA density or Kv4.2 expression, indicating that NRN1’s effects are mediated through IR rather than IGF-1R (Yao et al., 2012).

However, the relationship between NRN1 and IGF-1R signaling is not absent. In SCs exposed to hyperglycemic stress, NRN1 functioned as a downstream element of the IGF-1R-PI3K signaling pathway, mediating cellular rescue from apoptosis (Yan et al., 2018). This suggests that while NRN1 does not directly engage IGF-1R, significant intracellular crosstalk exists between NRN1 and IGF-1R-PI3K signaling networks, highlighting the complexity of neurotrophic factor interactions.

### 4.2 MEK–ERK and PI3K-Akt–mTOR cascades

Given NRN1’s classification as a neurotrophic factor, key intracellular signaling pathways associated with neuronal development and function represent logical targets for investigation. Treatment of cerebellar granule neurons with NRN1 resulted in increased phosphorylation of ERK1/2, Akt, and mTOR, implicating both the MEK–ERK and PI3K-Akt–mTOR cascades in NRN1’s downstream effects (Yao et al., 2018). The functional significance of these pathways was confirmed through pharmacological inhibition studies: selective inhibitors of MEK–ERK, PI3K, and mTOR significantly blocked NRN1-induced Kv4.2 expression and the associated enhancement of IA current density.

NRN1 exhibits distinct temporal signaling of its downstream pathways. ERK phosphorylation peaks quickly within 30 min and returns to baseline by 60 min. Conversely, Akt and mTOR phosphorylation are slower, peaking at 6 h and 24 h, respectively. This separation potentially indicates NRN1 manages both immediate responses and long-term cellular changes, enabling precise regulation of neuronal function over different time scales.

### 4.3 Calcium-calcineurin-NFAT signaling

Building on previous work demonstrating the importance of Ca^2+^ signaling in NRN1 gene transcriptional activation, researchers discovered that calcium-dependent pathways are also crucial for NRN1-mediated Kv4.2 expression. NRN1 treatment increases expression of L-type Ca^2+^ and the T-type Ca^2+^ channels, which enhances inward cellular Ca^2+^ currents (Yao et al., 2016). This calcium influx subsequently activates the calcineurin (CaN)-nuclear factor of activated T cells c4 (NFATc4) signaling axis.

The functional importance of this pathway was demonstrated through pharmacological intervention: both calmodulin (CaM) inhibitors and CaN inhibitors have effectively prevented NRN1-mediated transcriptional enhancement of Kv4.2. Notably, the successful disruption of NRN1-induced Kv4.2 expression with cyclosporine, a CaN inhibitor, provides strong evidence that NRN1 engages the Ca^2+^-CaN-nuclear factor of activated T cells c4 (NFATc4) axis to regulate Kv4.2 expression and IA current (Yao et al., 2016). Other groups have also investigated NRN1-related signaling pathways. These studies have yielded similar results, demonstrating that NRN1 activates the MAPK, ERK, PI3K-Akt, and Ca^2+^ pathways (Azuchi et al., 2018).

### 4.4 FGFR and notch pathway interactions

The structural characteristics of NRN1 as a GPI-anchored protein provide essential clues about its potential receptor interactions. GPI-anchored proteins preferentially associate with tyrosine kinase-coupled receptors within specialized membrane microdomains called lipid rafts (Trupp et al., 1996; Marler et al., 2008). Based on this principle, Shimada et al. proposed that NRN1 might activate downstream signaling through the fibroblast growth factor (FGF) receptor, a member of the tyrosine kinase receptor family (Shimada et al., 2016).

Experimental validation of this hypothesis came through studies of NRN1-overexpressing cultured hippocampal granule neurons. Treatment with FGF receptor inhibitors completely abolished NRN1-induced axonal branching, while siRNA-mediated knockdown of FGFR1 similarly inhibited NRN1-mediated axonal development (Shimada et al., 2016). These findings suggest that FGFR signaling represents another major pathway through which NRN1 promotes structural neuronal remodeling. In contrast to its activating effects on growth-promoting pathways, NRN1 appears to function as an inhibitor of Notch signaling, a pathway traditionally associated with limiting neurite extension and maintaining neural stem cell homeostasis (Zhang et al., 2017). Notch signaling operates through direct cell-to-cell contact, involving interactions between Notch ligands and receptors on neighboring cells. It plays crucial roles in the CNS and the development and maintenance of neural stem cells (Lathia et al., 2008; Lampada and Taylor, 2023).

Research also revealed that NRN1 interacts with neutralized, NEURL1, a ubiquitin ligase that regulates Notch signaling by controlling ligand endocytosis. By interfering with this process, NRN1 inhibits a critical step in Notch signaling activation, thereby suppressing overall Notch pathway activity and promoting neurite outgrowth (Zhang et al., 2017).

This inhibitory relationship has important developmental implications. The researchers speculated that as NRN1 levels naturally decline following CNS maturation, the NRN1-mediated restriction of Notch signaling is progressively lifted. This results in the cessation of neurite outgrowth and establishment of a regulated balance between neurite growth and retraction, ultimately maintaining appropriate CNS structure and size (Zhang et al., 2017).

### 4.5 Downstream effector proteins

Despite significant progress in mapping upstream signaling pathways, relatively little is known about NRN1’s downstream mechanistic targets and effector proteins. To address this gap, researchers developed a systematic approach for identifying potential downstream targets based on three rational criteria: (1) expression in brain regions enriched for NRN1, (2) functional relevance to neural excitability through promotion of outward potassium currents, and (3) established connections to neurite growth and development.

Using these selection criteria, researchers identified leucine-rich glioma inactivated 1 (LGI1) as a promising candidate downstream target. LGI1 is a secreted protein associated with synaptic transmission, neuronal excitability, and epilepsy when mutated. Experimental validation revealed that NRN1 enhances LGI1 expression in the hippocampus through NRN1-mediated histone deacetylase 5 (HDAC5) phosphorylation by protein kinase D (Lee et al., 2021).

This epigenetic mechanism represents an additional layer of NRN1’s regulatory complexity, demonstrating that its effects extend beyond traditional kinase-mediated phosphorylation cascades to include chromatin remodeling and transcriptional control. The NRN1-LGI1 pathway contributes to enhanced stress resilience and supports neurite growth and branching, identifying LGI1 as a functionally significant downstream effector molecule.

The successful identification of LGI1 as an NRN1 effector, achieved through rational selection criteria, demonstrates the validity of this approach and provides a framework for discovering additional downstream targets. This methodology offers a systematic alternative to unbiased screening approaches and may prove particularly valuable for identifying tissue-specific or functionally specialized effector proteins.

Independent studies have corroborated many of these findings, demonstrating that NRN1 consistently activates MAPK, ERK, PI3K-Akt, and Ca^2+^ pathways across different experimental systems and cell types (Shimada et al., 2016; Lee et al., 2021). This reproducibility across laboratories and experimental conditions strengthens confidence in these mechanisms and suggests that they represent core components of NRN1’s signaling repertoire.

**Figure 2:**
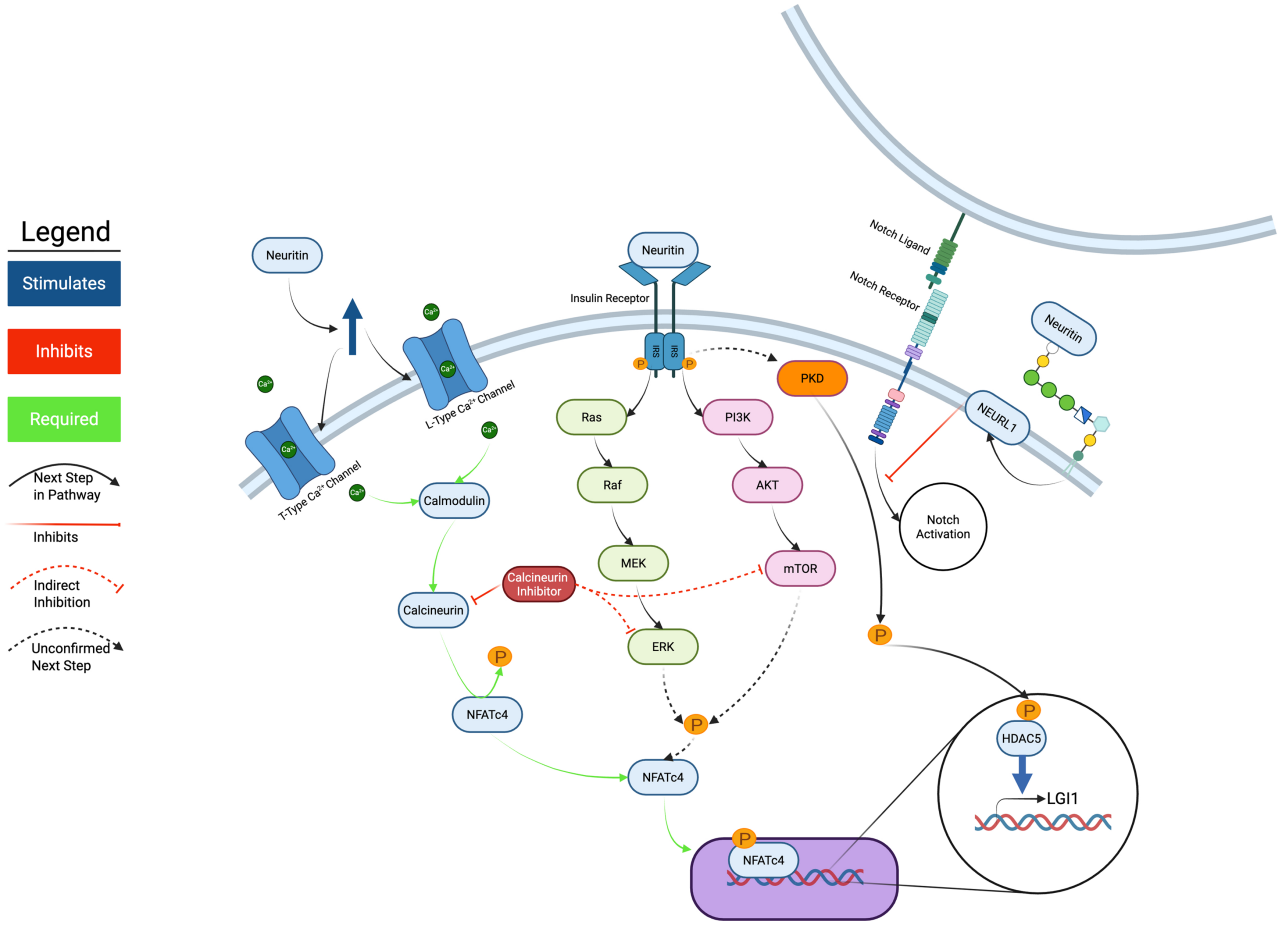
Current overview of NRN1 cellular signaling mechanisms. Abbreviations: CaM-Calmodulin, CaN-Calcineurin, NFATc4-Nuclear factor of activated T cells, MEK-Mitogen activated protein kinase, ERK-Extracellular-signal-regulated kinase, PI3K-Phosphoinositide 3 kinase, Akt-Protein kinase B, mTOR-mammalian target of rapamycin, NEURL1-Neuralized, PKD-Protein kinase D, HDAC5-Histone deacetylase 5. Created in BioRender.com.

The convergence of multiple signaling pathways downstream of NRN1, including IR activation, MEK–ERK and PI3K-Akt–mTOR cascades, calcium-calcineurin-NFAT signaling, FGFR engagement, and Notch inhibition, suggests a highly integrated signaling network. A simplified schematic of Nrn1’s complex signaling pathways and downstream targets is depicted in Figure 2. This complexity likely reflects NRN1’s diverse physiological roles and its ability to coordinate multiple aspects of neuronal function, from immediate changes in excitability to long-term structural remodeling.

Current evidence supports a model in which NRN1 functions as a multifaceted signaling molecule that engages diverse receptor systems and intracellular pathways, as can be seen in Figure 2. The identification of insulin receptor as a direct target, combined with evidence for FGFR interactions and the temporal dynamics of downstream kinase activation, begins to paint a picture of NRN1 as a coordinator of neuronal responses across multiple timescales and functional domains.

The development of systematic approaches for identifying downstream effector proteins, exemplified by the discovery of LGI1, provides a roadmap for future mechanistic studies. Understanding these terminal effectors will be crucial for comprehending how NRN1’s diverse signaling inputs are translated into specific physiological outputs.

## 5 NRN1: role and potential therapeutic application across neurodegenerative and other clinical conditions

NRN1 is being explored as a potential therapy across specialties through leveraging its mechanistic processes to hinder the driving pathophysiological forces behind clinical conditions such as glaucoma, AD, and stroke. A complete table highlighting NRN1’s mechanistic processes across clinical conditions is depicted in Tables 1, 2.

**Table 1:** Summary of Nrn1’s mechanistic role and clinical application across neurodegenerative clinical conditions.

Clinical condition	Clinical application	Mechanism of action	Key findings	References
Glaucoma	Neuroprotection	- ECM remodeling (↓ fibronectin, ↓ collagen IV) - Anti-apoptotic signaling (↑ Bcl-2, ↓ caspase-3) - RGC preservation - Functional protection (improved ERG)	- Reduced axonal degeneration - Preserved retinal function with elevated IOP - Enhanced RGC survival - Improved structural integrity	Sharma et al. (2015), Azuchi et al. (2018), Hameed et al. (2024)
Optic neuropathies	Neuroprotection	- RGC survival promotion - Neurite outgrowth enhancement - Regeneration marker upregulation - Axonal transport facilitation	- Enhanced RGC survival - Increased Gap43 expression - Preserved visual function - Active axonal transport across lesions	Sharma et al. (2015), Azuchi et al. (2018)
Diabetic neuropathy	Peripheral neuroprotection	- NGF-mediated NRN1 upregulation - SC survival enhancement - Anti-apoptotic (↑ Bcl-2, ↓ caspase-3) - Neurite outgrowth protection	- Restored NRN1 levels with NGF treatment - Improved SC survival - Enhanced axonal regeneration capacity	Karamoysoyli et al. (2008), Xi et al. (2020)
Alzheimer’s disease	Cognitive resilience and anti-neuroinflammation	- Synaptic integrity maintenance - Anti-amyloid-beta toxicity - miRNA-mediated regulation (miR-199a) - Anti-apoptotic pathways - NF-κB pathway suppression - Cytokine reduction (↓ IL-1β, ↓ TNF-*α*) - Hippocampal expression normalization	- Positive correlation with preserved cognition - Protection against Aβ-induced toxicity - Downregulation by miR-199a in AD brain - Dose-dependent cognitive rescue in STZ model - Reduced neuroinflammatory signaling - Suppressed cytochrome c release - Decreased cleaved caspase-3 levels	Song et al. (2020), Yu et al. (2024), Hurst et al. (2023), Kazemi et al. (2025)
Age-related hearing loss	Neuroprotection, ototoxicity protection	- Spiral ganglion neuron protection - Anti-senescence mechanisms - Shielding against time induced cell death - Anti-oxidative effect - Autophagy stimulation - Cyto-protective - Cellular integrity protection	- Prevented cochlear hair cell loss - Increased nerve fiber density - Preserved auditory thresholds (24–36 weeks) - Reduced reactive oxygen species production ex vivo - Protection against aminoglycoside damage - Reduced cisplatin-induced injury - Enhanced cochlear structure preservation	Huang et al. (2022), Fei et al. (2024), Wang et al. (2025)

**Table 2:** Summary of neuritin’s mechanistic role and clinical application across other clinical conditions.

Clinical condition	Clinical application	Mechanism of action	Key findings	References
Stroke	Neuroprotection	- Synaptic transmission enhancement - Safeguarding against demyelination - Synaptic vesicle protein maintenance - Behavioral recovery promotion - Anti-inflammatory signaling - Tissue preservation pathways	- Improved locomotor and neurologic function - Reduced lesion volume and edema - Myelin integrity preservation - Enhanced spontaneous excitatory postsynaptic currents frequency/amplitude - Preserved corpus callosum integrity - Decreased inflammatory markers	Lu et al. (2021), Xu et al. (2023), Xu et al. (2024)
Spinal Cord Injury	Neuroregeneration	- Biphasic Nrn1 expression pattern - Injury-to-repair transition facilitation - Growth factor coordination - Plasticity enhancement	- Initial downregulation post-injury - Strong upregulation during regeneration - Enhanced locomotor function - Improved axonal regeneration	Di Giovanni et al. (2005), Gao et al. (2016)
Neuropsychiatric disorders	Neuroregulation and genetic susceptibility	- Serotonergic circuit development - Prefrontal cortex axonal branching - Stress-induced expression suppression - Synaptic stabilization - Dorsolateral prefrontal cortex activation - Working memory network dynamics - Maintenance of fluid intelligence - SNPs (HAP678 GTT haplotype associated with early onset SZ)	- Impaired serotonergic branching in KO mice - Anxiety/depressive behaviors in Nrn1 KO - Neurobehavioral rescue with viral overexpression - Stress suppresses endogenous expression - Altered prefrontal activation in carriers in HAP678 GTT haplotype - Association with early-onset disease SZ - Cognitive dysfunction correlation	Son et al. (2012) , An et al. (2014), Almodovar-Paya et al. (2022), Shimada et al. (2024)
Melanoma	Biomarker	- Modulation of inflammatory tumor microenvironment - Treg expansion → coercion and constraining of immune effector cells → immune evasion - Angiogenesis promotion - Cell proliferation enhancement	- Elevated serum levels in patients - Enhanced tumor vascularization - Promoted melanoblast proliferation - Facilitated cell migration	Truzzi et al. (2008), Bosserhoff et al. (2017), Devitt et al. (2024), Zhang et al. (2025)

### 5.1 NRN1 in neuropsychiatric disorders

NRN1, characterized initially for its role in activity-dependent neural plasticity, has garnered increasing attention for its involvement in a range of neuropsychiatric and neurodegenerative conditions. Its neuroprotective functions and regulatory effects on synaptic architecture have prompted investigations into its role in disorders such as anxiety, depression, schizophrenia (SZ), and AD (Chandler et al., 2010; Fatjo-Vilas et al., 2016; Shimada et al., 2024). Emerging data from both clinical and preclinical studies suggest that NRN1 acts as a modulatory factor at the intersection of stress response, neuroinflammation, cognitive function, and circuit remodeling.

A pivotal study showed NRN1 regulates serotonergic circuit development and emotional behavior (Shimada et al., 2024). Using a NRN1 KO mouse model, authors found impaired axonal branching of serotonergic neurons in mood-related brain areas like the medial prefrontal cortex and basolateral amygdala. These deficits were linked to increased anxiety and depressive-like behavior, but viral overexpression of NRN1 rescued both the structural and behavioral issues. Additionally, exogenous stress reduced endogenous NRN1 expression, indicating a link between environmental stress and molecular plasticity.

NRN1 has also been implicated in schizophrenia. Genetic association studies have revealed that a specific NRN1 haplotype (HAP678 GTT) is associated with early-onset schizophrenia, indicating a possible role in disease vulnerability (Almodovar-Paya et al., 2022). Functional imaging studies have shown that carriers of this haplotype exhibit altered dorsolateral prefrontal cortex activation during working memory tasks, indicating a genotype–phenotype link between NRN1 variants and prefrontal cortex network dynamics, a region long implicated in cognitive dysfunction in SZ.

### 5.2 NRN1: link to cognitive resilience in Alzheimer’s disease

In the context of AD, NRN1 is emerging as a potential biomarker and mediator of cognitive resilience. A proteomic study of postmortem cortical tissues from individuals with asymptomatic AD, symptomatic AD, and cognitively normal controls identified NRN1 as a protein whose expression positively correlated with preserved global cognition (Hurst et al., 2023). This suggests that NRN1 may buffer against clinical manifestation despite the presence of neuropathologic features. Complementary findings from another study identified NRN1 among a panel of cortical proteins associated with cognitive resilience (Hurst et al., 2023). Mechanistically, NRN1 appears to exert protective effects against amyloid-beta–induced toxicity, likely through modulation of synaptic integrity and anti-apoptotic signaling pathways.

The regulation of NRN1 expression in AD may itself be aberrant. MicroRNA-mediated suppression of NRN1 has been implicated in the pathogenesis of AD. Elevated levels of miR-199a in the AD brain have been shown to downregulate NRN1 expression, suggesting a role for dysregulated miRNA-mediated NRN1 signaling in impaired synaptic maintenance and neurodegeneration (Song et al., 2020).

Experimental interventions targeting NRN1 signaling offer promising therapeutic prospects. In animal models of both chronic stress and AD, viral-mediated overexpression of NRN1 has produced robust neuroprotective effects, including the prevention of dendritic spine loss, reversal of depressive-like behavior, and restoration of cognitive function (Son et al., 2012; An et al., 2014; Choi et al., 2014; Lee et al., 2021; Song et al., 2025). These results underscore the versatility of NRN1 as a modulator of neural integrity across diverse pathological conditions.

A recent study demonstrated the therapeutic potential of exogenous NRN1 in a streptozotocin (STZ)-induced rat model of AD (Kazemi et al., 2025). Administration of NRN1 at graded doses (0.5, 1.0, and 1.5 μg) significantly reversed STZ-induced cognitive deficits in both the novel object recognition and Morris’s water maze tasks. The Morris water maze results, in particular, highlighted NRN1’s potential in reversing learning deficiencies. Notably, NRN1 treatment reduced escape latency times in the Morris water maze, approaching levels found in the control animals. Given the dose-dependent effect of NRN1 on STZ-induced neurological issues, a gene expression analysis was conducted at the 1.5 μg treatment level, which normalized hippocampal NRN1 expression and suppressed neuroinflammatory signaling, as evidenced by reduced NF-κB activation and downstream cytokines IL-1β and TNF-*α*. Moreover, NRN1 attenuated markers of apoptosis, including the release of cytochrome c and the levels of cleaved caspase-3. These findings provide a mechanistic bridge between NRN1’s cognitive effects and its ability to modulate key pathways in inflammation and neuronal survival (Kazemi et al., 2025).

Collectively, this growing body of evidence positions NRN1 as a critical node in the regulation of synaptic architecture, stress resilience, and cognitive maintenance. Its multi-dimensional role across neurodevelopmental, affective, and degenerative disorders not only enhances our understanding of pathophysiology but also opens the door for novel therapeutic approaches aimed at restoring or augmenting NRN1 signaling.

### 5.3 NRN1: a promising neuroprotective agent in glaucoma

Ophthalmology remains at the forefront of innovation in neuroprotection and nerve regeneration, driven by the urgent need to prevent irreversible vision loss. Among optic neuropathies, glaucoma remains the leading cause of irreversible blindness worldwide and serves as a primary model for studying neuroprotective strategies (Shan et al., 2024).

**Figure 3:**
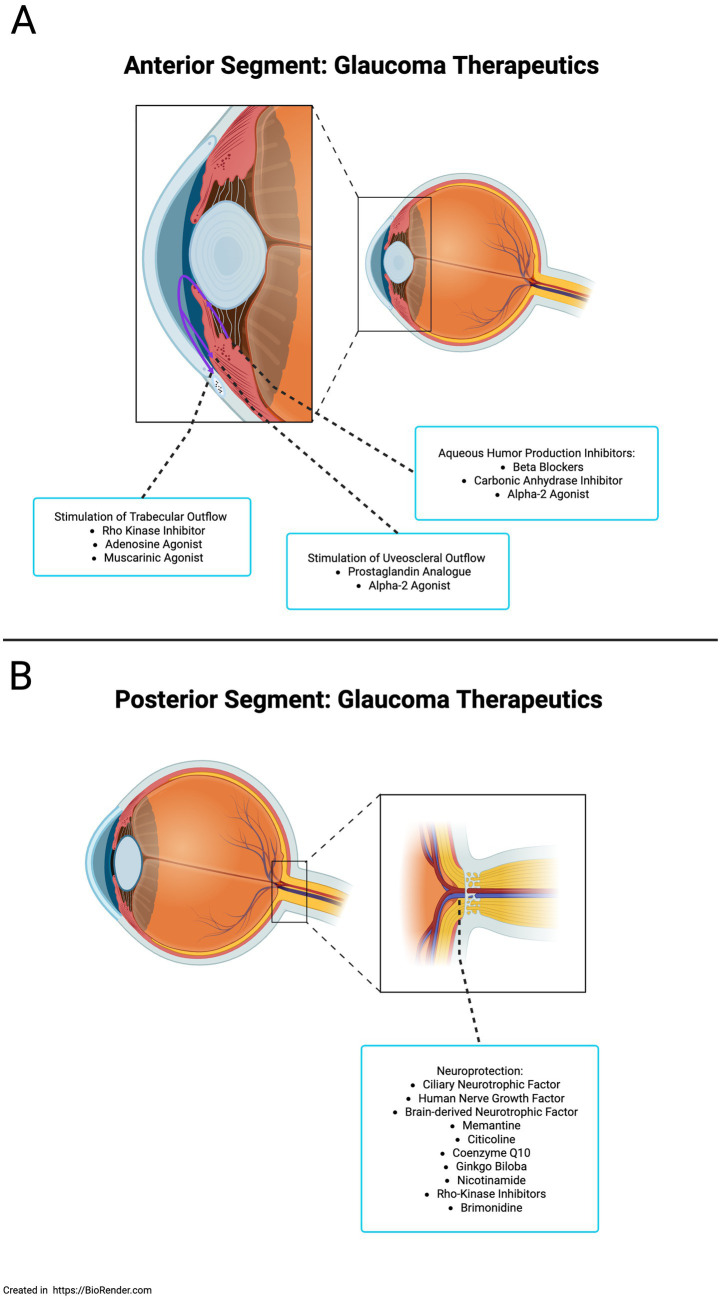
**(A)** Glaucoma therapeutics: anterior segment strategies focus on lowering IOP. **(B)** Glaucoma therapeutics: posterior segment strategy focuses on neuroprotection. Created in BioRender.com.

Glaucoma is characterized by the progressive degeneration of RGCs and their axons, leading to optic nerve damage and gradual vision loss. Elevated intraocular pressure (IOP) is the most significant modifiable risk factor for glaucoma. Consequently, current management strategies focus predominantly on IOP-lowering therapies, as can be seen in Figure 3A, which are effective in slowing disease progression for many patients (Weinreb et al., 2014). However, a considerable subset of individuals continues to experience visual decline despite achieving target IOPs. Especially those with normal-tension glaucoma, highlighting the need for adjunctive neuroprotective interventions (Anderson et al., 2001; Lee et al., 2020).

In response to this unmet clinical need, the field has shifted toward developing neuroprotective therapies that directly preserve RGCs and support the health of the optic nerve as can be seen in Figure 3B. Within this emerging therapeutic landscape, NRN1 has garnered attention for its potential role as a novel neurotrophic factor with applications in glaucoma (Lee et al., 2024; Wang et al., 2024). Expression profiling of glaucomatous donor retinas reveals significantly reduced levels of NRN1 and RGC-specific markers compared to healthy controls, suggesting a potential link between NRN1 deficiency and the neuroinflammatory environment characteristic of glaucomatous optic neuropathy (Hameed et al., 2024).

Experimental studies in animal models and ex vivo systems have substantiated NRN1’s neuroprotective and regenerative effects on RGCs. In both optic nerve injury and glaucoma models, NRN1 has demonstrated the capacity to support the survival of RGCs and their neurite outgrowth, highlighting its therapeutic relevance (Sharma et al., 2015; Azuchi et al., 2018).

Recent work has utilized a sophisticated ex vivo model, the Translaminar Autonomous System (TAS) model, to simulate the pressure differentials across the posterior segment of the eye, as observed in glaucoma (Hameed et al., 2024). This model allows precise control over IOP and provides a physiologically relevant platform for testing neuroprotective agents in human donor eyes.

In a recent study utilizing this TAS model, donor globes without prior ocular disease were subjected to either normal or elevated IOP for 7 days and perfused with neurobasal media, with or without recombinant NRN1 (Hameed et al., 2024). In the high IOP condition, NRN1-treated eyes demonstrated marked neuroprotective effects across multiple endpoints.

Results revealed that NRN1 treatment led to significant reductions in fibronectin and collagen IV accumulation, which are typically elevated under glaucomatous conditions and contribute to optic nerve head remodeling. There was a notable decrease in the expression of apoptotic markers, suggesting enhanced neuronal survival mechanisms. Improved RGC survival was indicated by a significant increase in the expression of RNA-binding protein with multiple splicing, a highly specific marker of RGCs, in the NRN1-treated group under high IOP, indicating enhanced RGC preservation. Functional assessment using electroretinography demonstrated improved retinal signaling in the NRN1-treated group under high IOP conditions, underscoring the translational significance of these findings.

**Figure 4:**
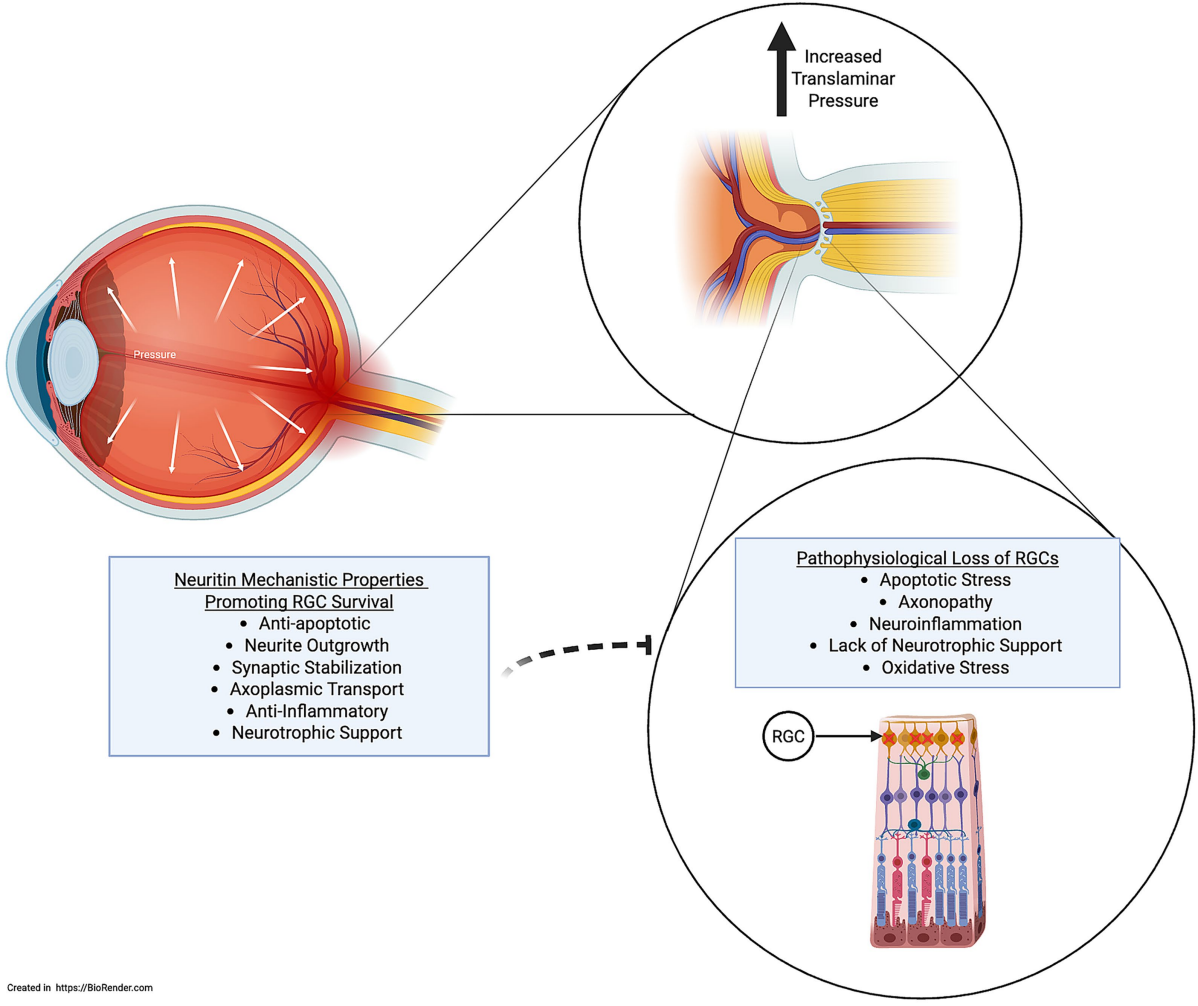
An increased translaminar pressure gradient drives the pathophysiological loss of RGCs. NRN1 inhibits the loss of RGCs through neuroprotective mechanisms.

Collectively, these results support the therapeutic potential of NRN1 as a neuroprotective agent in glaucoma. Figure 4 depicts NRN1’s mechanistic properties as a potential therapeutic strategy to target RGC loss in glaucoma. Notably, the multi-faceted benefits observed, ranging from extracellular matrix modulation and anti-apoptotic signaling to functional preservation of visual circuitry, position NRN1 as a compelling candidate for further development. Beyond glaucoma, similar mechanisms may also apply to other optic neuropathies, such as anterior ischemic optic neuropathy, which shares overlapping pathways of RGC injury and neuroinflammation.

While these findings are promising, additional *in vivo* validation, pharmacokinetic optimization, and clinical trials will be essential to advance NRN1-based therapies toward clinical application. Nevertheless, the accumulating evidence situates NRN1 at the intersection of molecular neuroprotection and translational ophthalmology, offering hope for novel strategies to preserve vision in patients at risk of progressive optic neuropathies.

### 5.4 NRN1: emerging applications in auditory neuroprotection

While NRN1 has gained attention for its neuroprotective effects in the visual system, growing evidence suggests that it may also have therapeutic value in the auditory system. Recent studies highlight NRN1’s protective role against both pharmacologically induced and age-related cochlear degeneration, positioning it as a promising candidate for future interventions aimed at mitigating sensorineural hearing loss (Huang et al., 2022; Fei et al., 2024; Wang et al., 2025).

A seminal study by Huang and colleagues demonstrated that NRN1 plays a protective role in maintaining cochlear health, particularly in preserving hair cells and spiral ganglion neurons, two key structures involved in auditory signal transduction (Huang et al., 2022). Initially, their work focused on the ability of NRN1 to counteract drug-induced ototoxicity. In experimental models of hair cell and spiral ganglion neuron degeneration by aminoglycoside and cisplatin, *Nrn1* overexpression significantly reduced structural damage and preserved cellular integrity (Huang et al., 2022). This foundational research provided compelling evidence for NRN1’s direct cytoprotective action in the inner ear.

In that investigation, a *Nrn1* knock-in mouse model was utilized, and cochlear integrity and auditory thresholds were monitored over 48 weeks. Their longitudinal analysis revealed that *Nrn1* overexpression prevented significant loss of cochlear hair cells, especially in the basal and middle cochlear turns, which are more vulnerable to age-related degeneration. They noted increased nerve fiber density in supporting cells and spiral ganglion neurons, indicating enhanced neuronal connectivity and reduced axonal loss. Auditory thresholds were maintained in early and mid-life stages. At 24 and 36 weeks, mice with *Nrn1* overexpression had significantly lower hearing thresholds compared to age-matched controls, indicating improved auditory function (Huang et al., 2022).

Interestingly, by 48 weeks of age, the auditory thresholds of the *Nrn1* overexpression group were no longer significantly different from those of the control group, despite histological evidence of preserved cochlear structure and nerve fiber density (Huang et al., 2022). This finding suggests that while NRN1 effectively maintains cellular and structural integrity in the cochlea, age-related changes in auditory processing may involve additional mechanisms not entirely mitigated by NRN1 alone. These results raise important questions about the temporal window of NRN1 efficacy and the potential need for combinatorial therapies in the long-term preservation of auditory function.

To further elucidate NRN1’s protective effects in the cochlea, researchers turned to ex vivo studies using neonatal mouse cochlear basilar membranes. In this model, oxidative stress and senescence were induced using d-galactose, a sugar known to mimic aging-like cellular damage. When cultured cochlear basilar membranes were treated with exogenous NRN1, researchers observed a significant reduction in reactive oxygen species production and a decrease in the expression of apoptotic markers, including cleaved caspase-3 and Bax (Wang et al., 2025). This suggests a role for NRN1 in reducing oxidative stress and indicates improved cell survival under stress conditions.

Ex vivo findings corroborate the *in vivo* data and highlight NRN1’s potential as an anti-senescence and anti-apoptotic factor in the inner ear (Wang et al., 2025). By targeting the fundamental mechanisms of cochlear aging (oxidative stress, apoptosis, and structural degeneration), NRN1 offers a novel therapeutic strategy for preserving auditory function.

Taken together, these studies provide compelling preclinical evidence supporting the role of NRN1 in auditory neuroprotection. Whether delivered genetically or pharmacologically, NRN1-based interventions may have future applications in preventing or slowing the progression of age-related hearing loss and ototoxicity-induced auditory damage. Further investigation is needed to optimize delivery methods, understand long-term functional outcomes, and explore synergistic effects with other protective agents or regenerative therapies.

### 5.5 NRN1: a neuroprotective candidate in intracranial hemorrhage and ischemic stroke

Stroke remains a leading cause of adult disability and death worldwide, with limited therapeutic options aimed at promoting neuronal survival and recovery following injury. In both hemorrhagic and ischemic stroke, emerging data position NRN1 as a promising neuroprotective agent due to its multifaceted role in supporting synaptic function, reducing neuroinflammation, and enhancing tissue integrity (Zhao et al., 2017; Lu et al., 2021).

Recent investigations into intracerebral hemorrhage (ICH) have highlighted NRN1’s restorative capacity in animal models (Wan et al., 2020; Lu et al., 2021). In a study using adeno-associated virus-mediated overexpression of NRN1 in a murine model of ICH, researchers observed multiple beneficial effects (Lu et al., 2021).

*Nrn1* overexpression in mice exhibited significantly enhanced locomotor function compared to control ICH mice, indicating functional neurologic recovery. Histological analysis revealed decreased lesion volume, reduced cerebral edema, and diminished neuronal death in the perihematomal region. Electrophysiological recordings demonstrated marked improvements in synaptic transmission, including increased frequency and amplitude of spontaneous excitatory postsynaptic currents, elevated action potential firing rates, and restoration of delayed action potential onset, which is commonly disrupted following hemorrhagic injury. Myelin integrity was significantly improved in the corpus callosum and striatum, as evidenced by increased Luxol fast blue optical density (Lu et al., 2021). These findings suggest that NRN1 supports remyelination or reduces demyelination in injured tissue. Synaptic maintenance was validated using immunostaining for synapsin-1, a presynaptic vesicle protein that was notably elevated in the NRN1-treated group, consistent with preserved synaptic health (Lu et al., 2021). These findings underscore NRN1’s capacity not only to mitigate the immediate consequences of hemorrhagic insult but also to preserve essential components of neural circuitry.

From a translational perspective, a recent clinical study examined serum NRN1 levels in patients with spontaneous ICH. The study found that serum NRN1 levels were significantly elevated in patients with ICH compared to healthy controls. Their results also showed that higher circulating NRN1 levels correlated with increased hematoma volume, more severe neurological deficits at presentation, and worse outcomes at 90 days post-stroke (Xu et al., 2024). These findings suggest that endogenous upregulation of NRN1 may represent a reactive, injury-induced compensatory response. Consequently, NRN1 may serve as a biomarker for stroke severity and a potential predictor of long-term prognosis, although its precise pathophysiologic and diagnostic roles require further investigation.

In models of ischemic stroke, particularly those involving middle cerebral artery occlusion, NRN1 shows significant neuroprotective properties. A recent rat study found that overexpression of NRN1 led to a substantial reduction in infarct volume, indicating the preservation of brain tissue following ischemic injury. The decreased infarct volume was accompanied by improved neurologic function, as measured by standard neurobehavioral scoring systems. Additionally, NRN1 displayed an anti-chemotactic function, reducing markers of neuroinflammation, including the downregulation of key inflammatory cytokines in ischemic brain regions (Xu et al., 2023). These findings suggest that NRN1 may counteract the excitotoxic and inflammatory cascades that typify cerebral ischemia, thereby supporting its candidacy as a therapeutic agent.

Taken together, these preclinical and clinical findings position NRN1 as a multipotent neuroprotective factor in stroke. In both hemorrhagic and ischemic contexts, NRN1 promotes neuronal survival, maintains synaptic and axonal integrity, supports myelin preservation, and may modulate inflammatory responses. In contrast, elevated endogenous NRN1 levels may reflect disease severity; the therapeutic delivery of NRN1 via gene therapy or recombinant protein warrants further investigation in translational and clinical settings. Its dual potential as both a biomarker and an interventional target represents an exciting frontier in stroke neurobiology.

### 5.6 NRN1: a novel therapeutic target in diabetic neuropathy

Diabetic neuropathy is a common and debilitating complication of both type 1 and type 2 diabetes mellitus, affecting up to 50% of patients over their lifetime. Characterized by progressive axonopathy, sensory deficits, and chronic pain, the pathophysiology of diabetic neuropathy includes chronic hyperglycemia-induced oxidative stress, neuroinflammation, and dysregulated neurotrophic signaling (Sandireddy et al., 2014; Roman-Pintos et al., 2016; Msheik et al., 2022). These mechanisms lead to the degeneration of sensory and motor axons, as well as the loss of Schwann cell (SC) integrity, ultimately impairing peripheral nerve function. In this context, NRN1 has emerged as a promising candidate for therapeutic intervention. Its known roles in neuronal growth, axonal sprouting, synaptic plasticity, and cell survival align well with the pathological features observed in diabetic neuropathy. The convergence of oxidative injury and deficient neurotrophic support in diabetic nerve tissue makes NRN1 a relevant therapeutic strategy.

A pivotal study using a STZ-induced diabetic rat model demonstrated that diabetes markedly reduces NRN1 expression in dorsal root ganglia and sciatic nerve, which are key anatomical structures affected in peripheral neuropathy (Karamoysoyli et al., 2008). Notably, treatment with NGF restored NRN1 levels in these tissues, suggesting that the neurotrophic deficit in diabetes contributes to suppressed NRN1 signaling and that upstream neurotrophins may regulate NRN1 expression. This restoration suggests that NRN1 may act as a downstream effector of broader neurotrophic pathways.

Further supporting NRN1’s therapeutic potential, a separate study explored its direct effects on diabetic SCs, which are essential for peripheral nerve myelination and regeneration. In this study, exogenous application of NRN1 significantly improved survival of cultured SC from diabetic rats. NRN1 treatment altered the apoptotic balance by upregulating the anti-apoptotic protein Bcl-2 and downregulating the pro-apoptotic effector cleaved caspase-3. This intervention preserved SC morphology and enhanced the capacity for neurite outgrowth, indicating improved trophic support for axonal regeneration (Xi et al., 2020). Collectively, these findings position NRN1 not only as a survival factor for glial cells but also as a potential modulator of axonal repair and a mediator of the cellular stress response in diabetic peripheral nerves.

Given that diabetic neuropathy often resists conventional glycemic control strategies and current treatments are essentially symptomatic, there is an urgent need for disease-modifying therapies that can promote nerve regeneration and neuroprotection. NRN1’s emerging profile in diabetic neuropathy expands its known function beyond the central nervous system and into the realm of peripheral nerve regeneration. NRN1’s ability to counteract hyperglycemia-induced neurodegeneration, regulate apoptotic signaling, and promote structural regeneration highlights its translational promise as a therapeutic intervention. Further investigation into delivery mechanisms, dose–response relationships, and long-term outcomes will be essential to translating these promising preclinical findings into clinical applications.

## 6 NRN1: a potential cancer marker and therapeutic target

Although NRN1 has been extensively characterized for its roles in neurodevelopment and neuroregeneration, emerging evidence indicates that its functions extend beyond the nervous system, particularly implicating it in cancer biology as it is associated with 13 human cancers and could function as a biomarker or even a therapeutic target (Dong et al., 2018). Growing research suggests that NRN1 positively influences several hallmarks that drive malignancy and metastasis, such as cell proliferation, migration, immune modulation, and angiogenesis. This oncogenic relevance is especially notable in tumors of neural crest origin, such as melanoma, given the shared embryonic lineage between melanocytes and neural crest derivatives.

Melanocytes, the cells from which melanoma arises, originate from the neural crest, a transient embryonic structure that gives rise to diverse cell types, including neurons, glial cells, and melanocytes. Due to this common lineage, it is biologically plausible that neurotrophic factors, such as NRN1, have a significant effect on melanocytic tumors. Studies utilizing melanoma cell lines have demonstrated that NRN1 promotes melanoblast proliferation, enhances cell–cell adhesion, and facilitates cell migration and angiogenic processes. All of which are critical for tumor growth and metastatic dissemination (Truzzi et al., 2008; Bosserhoff et al., 2017). These findings suggest that NRN1 may mainly serve in a beneficial context as a developmental growth factor, however, in the case of cancer, it serves as a tumor-promoting molecule due to its role with cell migration and proliferation.

Clinically, serum NRN1 levels are significantly elevated in patients with melanoma compared to those with non-melanoma skin cancers (Bosserhoff et al., 2017). This correlation suggests that circulating NRN1 may function as a novel biomarker for melanoma diagnosis or prognosis. Its systemic presence indicates potential utility in non-invasive cancer monitoring, which could complement existing diagnostic tools. However, larger, prospective studies are necessary to validate NRN1’s effectiveness as a reliable biomarker and to understand its dynamics during disease progression.

Recent mechanistic studies have further elucidated the role of NRN1 in oncogenic signaling pathways. Notably, NRN1 has been shown to activate Signal Transducer and Activator of Transcription 3 (STAT3)-dependent pathways, leading to the transcription of genes involved in cell survival, proliferation, and immune evasion (Devitt et al., 2024). Since constitutive STAT3 activation is a well-established driver of tumor progression in various cancers, these findings position NRN1 as a potential upstream regulator of this oncogenic pathway. Targeting NRN1 could offer a novel therapeutic approach to inhibit STAT3-driven tumor growth.

In addition to its effects on tumor cell intrinsic pathways, NRN1 appears to modulate the tumor microenvironment by enhancing immune evasion mechanisms. Recent work revealed that NRN1 promotes the expansion and suppressive function of Tregs, which inhibit cytotoxic T lymphocyte responses and facilitate immune escape (Zhang et al., 2025). Significantly, blockade of NRN1 with specific monoclonal antibodies disrupted this immunosuppressive axis, restoring anti-tumor immunity. When combined with immune checkpoint inhibitors such as anti-PD-1 antibodies, NRN1-targeted therapy produced synergistic anti-tumor effects, suggesting that NRN1 inhibition could enhance the efficacy of existing immunotherapies.

Furthermore, consistent with its known roles in neurovascular development, NRN1 exerts potent pro-angiogenic effects within the tumor microenvironment. By promoting neovascularization, NRN1 supplies tumors with the necessary nutrients and oxygen for continued growth and metastatic potential. Consequently, strategies targeting NRN1 could simultaneously impair tumor angiogenesis and modulate immune responses, representing a promising dual-action therapeutic approach in highly vascularized and immunologically active tumors.

In summary, these diverse findings establish NRN1 as a multifunctional molecule with significant implications in oncology. Its capacity to promote tumor cell proliferation, activate oncogenic signaling pathways such as STAT3, facilitate immune suppression, and stimulate angiogenesis underscores its active participation in tumor biology. Future research should aim to delineate the full spectrum of NRN1’s molecular targets, optimize its potential as a biomarker, and develop targeted therapies that could be integrated into personalized cancer treatment regimens. Given its multifaceted influence on tumor progression, NRN1 represents a promising candidate for novel diagnostic and therapeutic strategies in the evolving landscape of precision oncology.

## 7 Discussion

The comprehensive examination of NRN1’s molecular mechanisms, neuroprotective properties, and diverse physiological functions presented in this review converges on a compelling opportunity for therapeutic innovation in neurodegenerative diseases. While there are still mechanistic gaps, the extensive documentation of NRN1’s protective and regenerative effects offers a solid foundation for ongoing investigation.

The current knowledge of NRN1’s complex mechanistic foundations provide crucial insights for therapeutic efficacy in neurodegenerative diseases. The protein’s engagement of multiple neuroprotective pathways, including IR activation, PI3K-Akt–mTOR cascades, calcium-calcineurin-NFAT signaling, FGFR-mediated growth promotion, and inhibition of mitochondrial stress-induced apoptosis, creates a robust molecular foundation for sustained neuroprotection (Yao et al., 2016). This multi-pathway activation is particularly relevant in glaucoma, where RGC death involves diverse mechanisms, including excitotoxicity, oxidative stress, neuroinflammation, and loss of neurotrophic support. Some remaining mechanistic questions that should be addressed in future mechanistic studies include: What are the structural determinants of NRN1’s receptor interactions? How do different signaling pathways integrate to produce cell-type-specific responses? What additional downstream effectors mediate NRN1’s effects in different brain regions and developmental contexts? Answering these questions will be essential for developing targeted therapeutic strategies that harness NRN1’s regenerative and neuroprotective capabilities across neurological and systemic diseases.

In ophthalmology, research on neuroprotective treatments is actively being pursued. Similar to NRN1, preclinical studies of neurotrophic factors have demonstrated the neuroprotective benefits BDNF, CNTF, and NGF (Beykin et al., 2022; Goldberg et al., 2023). Currently, the methods and delivery systems required to translate these findings into clinical practice remain unclear. Other pharmacologic strategies have been explored to boost neuroprotection, but establishing effective therapies in clinical settings remains challenging, and clinical benefits are still uncertain (Nucci et al., 2018; Wang et al., 2024).

**Figure 5:**
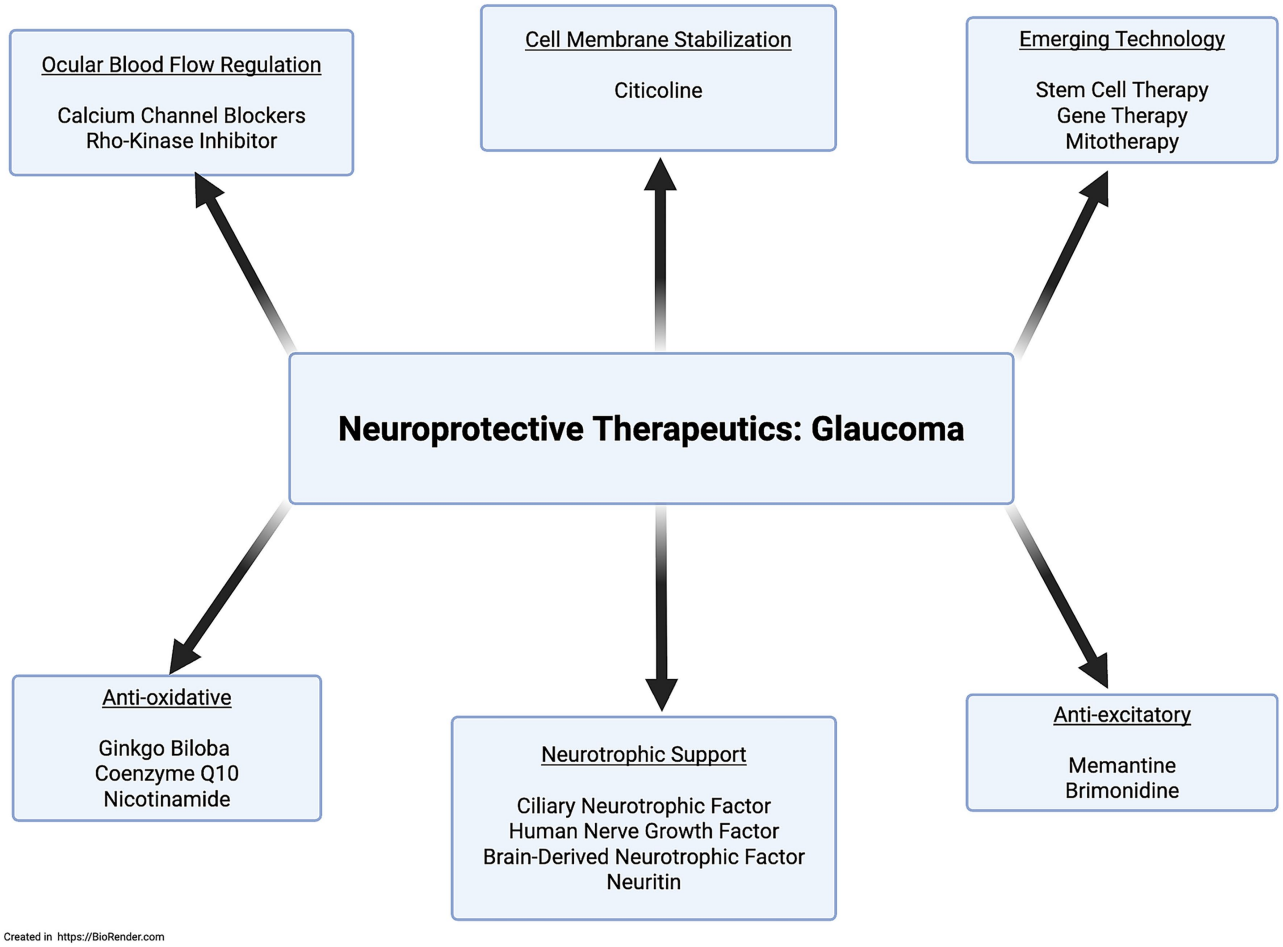
Mechanism-specific neuroprotective therapeutic agents.

Brimonidine significantly preserves the retinal nerve fiber layer compared to timolol, independent of IOP-lowering effects in ocular hypertension (Krupin et al., 2011; De Moraes et al., 2012). Larger trials are needed to confirm its neuroprotective benefits. Memantine, an anti-excitotoxic NMDA receptor antagonist with promising preclinical results, failed in large-scale clinical trials (Wang et al., 2024). *Ginkgo biloba*, used in traditional Chinese medicine for centuries for its antioxidant and mitochondrial stabilization shows no significant clinical benefit despite promising preclinical studies (Wang et al., 2024). Citicoline, a phosphatidylcholine precursor, supports membrane stability and neurotransmitter recycling; preclinical and early trials show neuroprotective effects in glaucoma, with a Phase III trial ongoing (Virno et al., 2000; Adibhatla et al., 2001; Rejdak et al., 2003; Parisi et al., 2008; Rossetti et al., 2020; Bhartiya, 2022). Nicotinamide, supporting mitochondrial health by replenishing NAD, shows decreased plasma levels in glaucoma patients. Clinical trials are promising, with early results indicating visual benefits; larger studies are ongoing (Nucci et al., 2018; Bhartiya, 2022; Wang et al., 2024). Additional neuroprotective agents are shown in Figure 5.

Given the involvement of the mTOR and PI3K/Akt pathways in NRN1’s downstream effects, it’s reasonable to consider that other ligands activating these pathways might also offer neuroprotective benefits. One such ligand is insulin, which activates the mTOR pathway. Research shows insulin supports neuronal survival, and abnormal insulin signaling has been observed in glaucoma patients. Preclinical evidence indicates exogenous insulin administration promotes dendritic arborization and synaptic stabilization after axonal injury. However, clinical trials have not yet confirmed whether insulin eye drops can preserve RGCs or slow visual field loss. Resveratrol, a PI3K/Akt activator and antioxidant, also shows promise as a neuroprotective agent. Animal studies indicate resveratrol improves RGC survival and may slow glaucoma progression. Emerging technologies like stem cell therapy, gene therapy, and mitotherapy represent additional neuroprotective treatment avenues. However, they come with complicating factors of safety and ethical concerns in addition to difficulty standardizing preclinical success into clinical practice. As neuroprotective research advances, efforts focus on creating personalized treatments targeting the root causes, such as neurotrophic support deficits and cellular energy dysfunction (Nucci et al., 2018; Sulak et al., 2024; Wang et al., 2024). Figure 5 provides a simplified illustration of ongoing neuroprotective approaches.

NRN1 is uniquely positioned as it can target multiple pathophysiological drivers of glaucoma, including lack of neurotrophic support and aberrant intracellular signaling, ultimately addressing the critical endpoint of glaucoma’s pathophysiological process: the loss of RGCs. NRN1 offers a comprehensive approach to neuroprotection that extends beyond single-target therapies, positioning it as a disease-modifying agent capable of addressing the complex pathophysiology underlying glaucomatous damage.

Particularly compelling is NRN1’s demonstrated efficacy in the sophisticated TAS model, which replicates the pressure differentials characteristic of glaucoma using human donor eyes (Sharma et al., 2020). The observed preservation of RGC-specific markers, reduction in extracellular matrix deposition, and maintenance of retinal function under elevated IOP conditions provide robust preclinical evidence for NRN1’s therapeutic potential. These findings are further supported by the protein’s safety profile under normal IOP conditions, indicating a favorable therapeutic window for clinical application.

The transition from preclinical promise to clinical reality requires careful consideration of delivery mechanisms, dosing strategies, and patient selection criteria. Further exploration is necessary through *in vivo* studies to validate *in vitro* findings and to optimize pharmacokinetics to identify the most effective delivery system capable of crossing the aqueous and vitreous regions of the eye to reach the retina. Some potential delivery approaches are intravitreal injection of recombinant protein, gene therapy vectors for sustained local expression, or novel drug delivery systems that could provide controlled release within the eye. Furthermore, for NRN1 to serve as a sustainable vision-restoring therapy, it is crucial to ensure the health and functionality of interneurons such as bipolar and amacrine cells, as they play a vital role in supporting the health of the targeted RGCs. Without adequate support from interneurons, RGCs may struggle to reintegrate into the retinal photo cascade and fail to receive visual signals from photoreceptors. Overall, the accumulating evidence positions NRN1 at the intersection of molecular neuroprotection and translational ophthalmology, offering hope for novel strategies to preserve and potentially restore vision in patients at risk of progressive optic neuropathies.

While glaucoma represents the primary target for NRN1-based therapeutics in ophthalmology, its broad neuroprotective effects imply potential applications across the spectrum of optic neuropathies. Conditions such as anterior ischemic, traumatic, and hereditary optic neuropathies share RGC injury pathways that could benefit from NRN1. The protein’s demonstrated efficacy in models of optic nerve crush and its ability to promote axonal regeneration suggest promise for acute optic neuropathies, where rapid intervention could preserve vision.

Research on the therapeutic capability of NRN1 for age-related hearing loss encourages continued investigation in otolaryngology to characterize the mechanisms of neuroprotective and regenerative effects by targeting the fundamental mechanisms of cochlear aging, such as oxidative stress, apoptosis, and structural degeneration. Further understanding potential neuroprotective pathways could lead to innovative future treatments that preserve hearing.

NRN1’s neuroprotective properties can play a role in stroke treatment. The treatment of stroke primarily focuses on prevention through anticoagulation and lifestyle changes, while the role of neuroprotection remains largely underexplored (Kuriakose and Xiao, 2020). With NRN1’s remarkable ability to reduce infarct size and shift the inflammatory microenvironment, future research should continue to investigate NRN1’s ability to optimize the neural cytokine profile and ameliorate neuroinflammation to enhance stroke recovery. Furthermore, future studies should focus on NRN1 as a potential mediator of improving and restoring cognition and function post-stroke.

The future of AD treatment increasingly emphasizes neuroprotection. In AD, as well as in other neurodegenerative disorders, there is a progressive loss of neurons that leads to cognitive decline (Pekdemir et al., 2024). Neuroprotection to target AD offers a promising treatment approach that utilizes neural plasticity to reorganize neural networks, thereby improving cognition and enhancing the quality of life for AD patients. NRN1’s potential therapeutic impact is tied to its cognitive resiliency functions. Future research should identify potential *NRN1* mutations and key pathways through which NRN1 enhances cognition to better understand its neuroprotective and regenerative mechanisms for neuroprotective therapeutics and to establish NRN1 as a marker of cognitive resilience. While genome-based studies in AD patients could map harmful single-nucleotide polymorphisms, characterize mutation frequency, and gene expression levels.

Additionally, the reported evidence of NRN1 in neuropsychiatric disorders highlights its importance in psychiatric research. NRN1’s ability to counteract stress, along with its connections to serotonergic neural networks, emphasizes its potential for future treatments of anxiety and depressive disorders. While current research suggests that polymorphisms in the NRN1 gene are linked to SZ, future studies should establish its role as a genetic marker for SZ and clarify whether genetic differences among SZ patients could enable personalized neuroprotective therapies.

As cancer rates rise globally, the demand for innovative oncology therapeutics will increase. NRN1 could enhance personalized immunomodulatory treatment regimens, as a positive regulator of checkpoint inhibitors to maximize the immune system’s tumor destruction. With that said, overwhelming evidence indicates that NRN1 is an effective therapeutic target across many conditions, but it may serve as a double-edged sword in oncology therapeutics since NRN1 has been shown to possess pro-angiogenic properties. Future research targeting NRN1 in cancer therapeutics should further clarify its angiogenic properties while harnessing its immunomodulatory properties.

The overall evidence presented positions NRN1 as a transformative therapeutic opportunity in glaucoma and other neurodegenerative disease management, representing a shift from purely pressure-based interventions to comprehensive neuroprotective strategies. The protein’s combination of proven efficacy, well-characterized mechanisms, favorable safety profile, and diverse delivery options creates a compelling foundation for clinical development. As the field of ophthalmology continues to embrace neuroprotective approaches, NRN1 stands as a leading candidate to bridge the gap between scientific discovery and clinical innovation, offering new hope for preserving vision in patients facing the devastating consequences of glaucomatous optic neuropathy.

The convergence of molecular understanding, preclinical validation, and clinical need creates an unprecedented opportunity to advance NRN1-based therapeutics toward clinical reality. Success in this endeavor would provide new treatment options for neurodegeneration and also establish a paradigm for neuroprotective intervention that could transform the management of optic neuropathies and broader neurodegenerative conditions.

## Glossary

NRN1: Neuritin
CPG: Candidate Plasticity-Related Gene
CNS: Central Nervous System
GPI: Glycosylphosphatidylinositol
CaMK: Calmodulin-Dependent Protein Kinase
CaM: Calmodulin
CaN: Calcineurin
MAPK: Mitogen-Activated Protein Kinase
NMDA: N-methyl-D-aspartate
AMPA: *α*-Amino-3-hydroxy-5-methyl-4-isoxazolepropionic acid
BDNF: Brain-Derived Neurotrophic Factor
TrkB: Tropomyosin Receptor Kinase B
CRE: cAMP Responsive Element
KO: Knockout
WT: Wild Type
NGF: Nerve Growth Factor
RGC: Retinal Ganglion Cell
Tregs: Regulatory T Cells
AD: Alzheimer’s disease
SZ: Schizophrenia
STZ: Streptozotocin
IR: Insulin Receptor
IGF-1R: Insulin-Like Growth Factor Type 1 Receptor
FGFR: Fibroblast Growth Factor Receptor
IA: Potassium Current
NFATc4: Nuclear Factor of Activated T Cells
NEURL1: Neuralized 1
HDAC5: Histone Deacetylase 5
IOP: Intraocular Pressure
ICH: Intracerebral Hemorrhage
SC: Schwann Cell
STAT-3: Signal Transducer and Activator of Transcription 3
